# The complete mitochondrial genome sequence of *Stichopus variegatus* (Echinodermata: Holothuroidea) and phylogenetic studies of Echinodermata

**DOI:** 10.1080/23802359.2019.1669502

**Published:** 2019-09-25

**Authors:** Qiuhua Yang, Qi Lin, Jianshao Wu, Fuyuan Yang, Hui Ge, Denggao Qiu, Zhongqin Li, Zhen Lu, Shengkang Li, Chen Zhou

**Affiliations:** a Key Laboratory of Cultivation and High-Value Utilization of Marine Organisms in Fujian Province, Fisheries Research Institute of Fujian, Xiamen, Fujian, China;; b Guangdong Provincial Key Laboratory of Marine Biology, Marine Biology Institute, Shantou University, Shantou, Guangdong, China;; c Fisheries College, Engineering Research Center on Eel Modern Industrial Technology of Ministry of Education, Jimei University, Xiamen, Fujian, China

**Keywords:** *Stichopus variegatus*, mitochondrial genome, phylogenetic analysis

## Abstract

At present, there exist some confusing issues on the species classification and phylogeny in Echinodermata. In this study, we first determined and described the complete mitochondrial genome of *Stichopus variegatus*. The complete mitogenome sequence had a circular mapping molecular with the total length of 16,315 bp and contained 13 protein-coding genes, 2 rRNA genes, 22 tRNA genes, and a putative control region. To further validate the newly determined sequences, phylogenetic trees involving all the Holothuroidea and other Echinodermata species available in GenBank Database were constructed. These results would be used for the species identification and further phylogenetic studies of Echinodermata.

The Echinodermata is divided into sub-phylum of Pelmatozoa and Eleutherzoa by their lifestyles, containing five classes of Crinoidea, Asteroidea, Ophiuroidea, Echinoidea, and Holothuroidea (Liao 1997). *Stichopus variegatus*, also named as *Stichopus herrmanni*, is one of the high economic species of holothurians, which occurs in a wide range of shallow tropical habitats (Purcell et al. 2012). In this work, we first determined and described the complete mitochondrial genome of *S. variegatus* (GenBank accession no. MN128376). One *S. variegatus* individual was collected from Wenchang, Hainan Province of China (19°25′40″ N, 110°45′14″ E). The specimen was preserved in the Culture Collection of Sea cucumber at the Fisheries research institute of Fujian of China (specimen number: 2019011412) and stored at −80 °C for DNA isolation. Genomic DNA extraction, PCR amplification, sequencing, and annotation were performed according to the methods described by Yang et al. (2015).

The complete *S. variegatus* mitogenome is a circular DNA molecule with the length of 16,315 bp. The genomic organization is identical to that of the typical Holothuroidea ground pattern (Scouras et al. 2004; Uthicke et al. 2009; Perseke et al. 2010; Yang et al. 2019), including 13 protein-coding genes, 2 rRNA genes, 22 tRNA genes, and a putative control region. The overall base composition was calculated by MEGA 6 (MEGA Inc., Tampa, FL) (Tamura et al. 2011): 31.58% (A), 29.16% (T), 23.48% (C) and 15.78% (G) showing a bias toward A + T (60.74%), similar to other Echinodermata mitochondrial genomes (Fan et al. 2011). The 13 protein-coding genes encode 3784 amino acids in total. The most frequently used amino acid is Leucine (16.20%) while Cysteine acid (0.95%) is the least frequently used one. The *12S* rRNA was 842 bp in length and located between *tRNA-Phe* and *tRNA-Glu*, while the *16S* rRNA was 1601 bp and located in the typical position between *nad2* and *cox1* (Mu et al. 2018). The 22 tRNA genes were interspersed between rRNAs and protein-coding genes, with sizes ranging from 59 bp (*tRNA-Ser*^AGN^) to 72 bp (*tRNA-Leu*^CUN^). The putative control region was located between *tRNA-Thr* and *tRNA-Pro* genes with 777 bp in length. All the features mentioned above are similar to the typical Holothuroidea mitogenome (Shen et al. 2009; Fan et al. 2012; Xia et al. 2016).

To further validate the newly determined sequences, phylogenetic trees involving all the Holothuroidea and other Echinodermata species available in GenBank Database were constructed with 13 mitochondrial protein-coding genes located on the heavy strand. We performed bootstrap analyses (1000 replicates) for both weighting schemes to evaluate relative levels of support for various nodes in the phylogenies. A maximum-likelihood tree (Swofford 2002) of 25 species in the sub-phylum of Eleutherzoa, with two Pelmatozoa species as outgroup species, was constructed (Figure 1).

**Figure 1.**
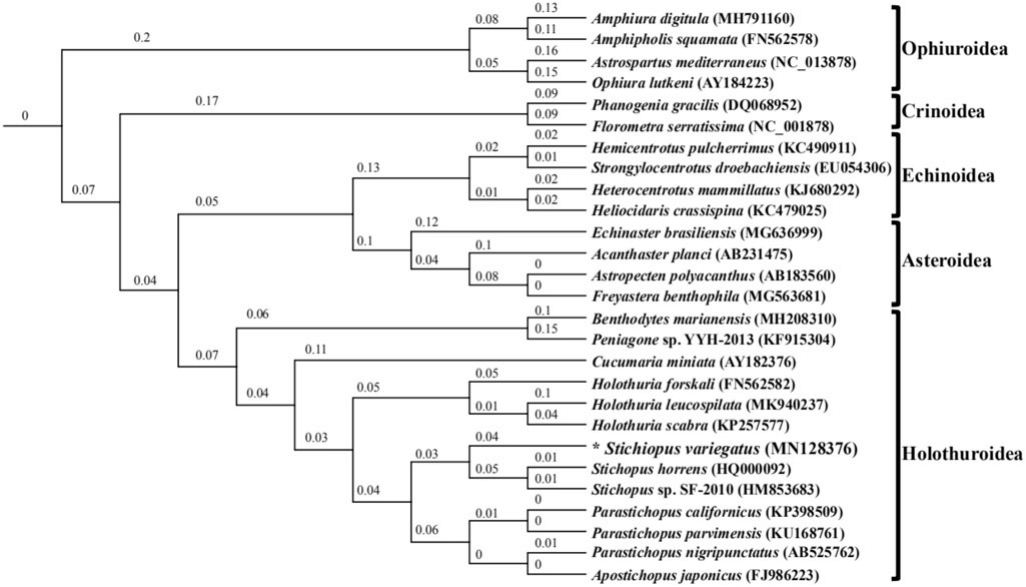
Maximum-likelihood tree inferred from 13 protein-coding genes of mitochondrial genomes of 25 Eleutherzoa species from four classes, using two Pelmatozoa species as outgroups. The bootstrap values are based on 1000 re-samplings. The number at each node is the bootstrap probability. The number after the species name in the brackets is the GenBank accession number. The asterisks before species names indicate newly determined mitochondrial genome in this paper.
